# Genomic region associated with run timing has similar haplotypes and phenotypic effects across three lineages of Chinook salmon

**DOI:** 10.1111/eva.13290

**Published:** 2021-09-01

**Authors:** Stuart C. Willis, Jon E. Hess, Jeff K. Fryer, John M. Whiteaker, Shawn R. Narum

**Affiliations:** ^1^ Hagerman Genetics Laboratory Columbia River Inter‐Tribal Fish Commission Hagerman ID USA; ^2^ Fishery Science Department Columbia River Inter‐Tribal Fish Commission Portland OR USA

**Keywords:** dominant, GWAS, haplotype, life history, migration, portfolio

## Abstract

Conserving life‐history variation is a stated goal of many management programs, but the most effective means by which to accomplish this are often far from clear. Early‐ and late‐migrating forms of Chinook salmon (*Oncorhynchus tshawytscha*) face unequal pressure from natural and anthropogenic forces that may alter the impacts of genetic variation underlying heritable migration timing. Genomic regions of chromosome 28 are known to be strongly associated with migration variation in adult Chinook salmon, but it remains unclear whether there is consistent association among diverse lineages and populations in large basins such as the Columbia River. With high‐throughput genotyping (GT‐seq) and phenotyping methods, we examined the association of genetic variation in 28 markers (spanning *GREB1L* to *ROCK1* of chromosome 28) with individual adult migration timing characteristics gleaned from passive integrated transponder recordings of over 5000 Chinook salmon from the three major phylogeographic lineages that inhabit the Columbia River Basin. Despite the strong genetic differences among them in putatively neutral genomic regions, each of the three lineages exhibited very similar genetic variants in the chromosome 28 region that were significantly associated with adult migration timing phenotypes. This is particularly notable for the interior stream‐type lineage, which exhibits an earlier and more constrained freshwater entry than the other lineages. In both interior stream‐type and interior ocean‐type lineages of Chinook salmon, heterozygotes of the most strongly associated linkage groups had largely intermediate migration timing relative to homozygotes, and results indicate codominance or possibly marginal partial dominance of the allele associated with early migration. Our results lend support to utilization of chromosome 28 variation in tracking and predicting run timing in these lineages of Chinook salmon in the Columbia River.

## 1 INTRODUCTION

It is generally understood that demographic or life‐history variations within a population, such as age at maturity or reproductive investments, act to buffer the population against short‐term environmental fluctuations (Hoelzel et al., 2019) and thereby provide stability in the ecosystem roles and services in which each species participates, commonly known as “portfolio effects” (Moore et al., 2014; Schindler et al., 2010, 2015). Many life‐history traits are known to exhibit significant heritability, suggesting not only that they are under selection and provide adaptive benefits within those populations, but also that they may be subject to influence by anthropogenic effects, such as habitat modification or fishery‐induced evolution (Cartwright et al., 2014; Heino et al., 2015; Waples & Audzijonyte, 2016). Moreover, the recognition that this genetic variation may be eroded by anthropogenic effects has prompted petitions for specific conservation management of vulnerable species that is directly tied to life‐history traits and associated genes (National Marine Fisheries Service, 2018). However, these conservation approaches need a clear understanding of the nature of the heritability and the strength of association to measurable markers (Heino et al., 2013; Waples & Lindley, 2018; Wray et al., 2013). This has added fuel to the search for genetic loci under selection or with strong association to known phenotypic variation in wild populations, a search which has been greatly empowered by the rapidly falling cost of sequencing and the many bioinformatic pipelines available to identify trait‐associated or outlier loci (Hoban et al., 2016).

Chinook salmon (*Oncorhynchus tshawytscha*) is a highly exploited Pacific salmon species that exhibits a number of heritable life‐history traits that have been used to identify and manage stocks for many years, in particular variation in adult migration patterns (Myers et al., 1998). These patterns have led to the identification of different “runs” of adult Chinook salmon commonly referred to by their peak season of migration (winter, spring, summer, fall), with early runs of fish entering freshwater several months prior to spawning in contrast to late runs that typically return in the fall and spawn shortly after (Ford et al., 2020; Quinn et al., 2016). While some rivers may host multiple runs, early‐returning fish tend to spawn higher in watersheds than late‐returning fish, and since many of these higher reaches are only accessible with high flow, this has led to the hypothesis that heritable earlier arrival was advantageous for populations accessing habitat that became inaccessible later in the year (Quinn et al., 2016). Different runs in the same river in coastal regions tend to be closely related relative to runs of the same phenotype in other areas, indicating either low‐to‐moderate ongoing gene flow in coastal populations (Hecht et al., 2015; Matala et al., 2011; Narum et al., 2008; O’Malley et al., 2013; Waples et al., 2004). In contrast, different runs of Chinook salmon in the interior Columbia River remain reproductively isolated and represent distinct lineages from those found in coastal regions including tributaries near the Columbia River estuary (Hecht et al., 2015; Narum et al., 2010; Waples et al., 2004). Previous management of runs of Chinook salmon has largely been based on the idea that most complex life‐history traits are polygenic and genetic variation for run timing should be broadly distributed across fish from both early and late runs (Thompson et al., 2019; Waples & Lindley, 2018; Waples et al., 2004).

Chinook salmon can be divided up into a number of hierarchical phylogeographic lineages (Beacham et al., 2006; Moran et al., 2013), and the populations in the Columbia River Basin exhibit three genetic lineages (Hecht et al., 2015; Narum et al., 2010; Waples et al., 2004). The “interior stream‐type” lineage (hereafter “iST”) is strongly divergent from the other two Columbia River Basin lineages (Hecht et al., 2015) known as “interior ocean‐type” (hereafter “iOT”; found exclusively east of the Cascade divide) and “Lower Columbia” (hereafter “LC”; found primarily in reaches nearest the estuary and coast). The LC and iOT lineages are more similar to one another than the iST lineage despite that the two interior lineages occur in greater sympatry.

Populations of Chinook salmon in the interior lineages are characterized by a narrower life‐history variation than the LC lineage (Healey, 1991; Myers et al., 1998). While iOT Chinook salmon exhibit both summer and fall runs, they exhibit outmigration generally as subyearlings. In contrast, iST Chinook salmon outmigrate as yearlings and show a more constrained return time, in spring to early summer. While the “stream‐type” and “ocean‐type” terminology apply poorly in coastal regions (Moran et al., 2013), they effectively describe interior lineages of the Columbia River and we include the “interior” designation for consistency with other literature on these Columbia Basin lineages and their life‐history variation (Koch & Narum, 2020; Moran et al., 2013; Narum et al., 2018). These life‐history variations have allowed managers to identify different stocks within the Columbia Basin prior to genetic identification, since only a subset of stocks were present during predefined periods (Myers et al., 1998). However, understanding genetic associations of run timing variation in Chinook salmon returning to the Columbia Basin provides another tool to apply for fisheries conservation and management (Waples & Lindley, 2018).

Several recent studies of genomic variation in Chinook salmon have identified a region of large effect for run timing on chromosome 28 that contains two genes, the human homologs for which are “*GREB1* Like retinoic acid receptor coactivator” (*GREB1L*) and “rho associated coiled‐coil containing protein kinase 1” (*ROCK1*; Narum et al., 2018; Prince et al., 2017). Both genes are understood to be involved in pathways affecting gonadal development, actin‐myosin contraction, and renal development and are expressed in renal and reproductive tissues (Brophy et al., 2017; De Tomasi et al., 2017; Mizuno et al., 2013; Nakagawa et al., 1996; Oviedo et al., 2011; Sanna‐Cherchi et al., 2017), and so could credibly be involved in reproductive maturation and environmental acclimation in anadromous salmonids. This gene region explained >70% of variance in run timing in some instances (Koch & Narum, 2020; Narum et al., 2018; Prince et al., 2017; Thompson et al., 2019) and may have partially dominant phenotypic expression, in which case it is unlikely that a late run could act as a refuge or reservoir for genetic variation to bolster a beleaguered early run (Koch & Narum, 2020; Thompson et al., 2019). This revelation has led some conservationists to petition for runs of Chinook salmon to be considered separate stocks in order to ensure that the full extent of life‐history variation is maintained, but as yet we do not have a complete understanding of the effects of these genes on run timing variation in different stocks, or the predictive value of the different molecular markers currently available (Ford et al., 2020; Waples & Lindley, 2018).

The large variation in life‐history suites of the three Columbia Basin Chinook salmon lineages make it challenging to predict the utility of chromosome 28 markers in predicting run timing. While a study using whole‐genome variation identified the same region of chromosome 28 in association with run timing in each of the lineages (Narum et al., 2018), it is as yet unclear whether markers in this region have the same predictive value. For example, in a study of hatchery‐spawned Chinook salmon and using categorial run timing phenotypes for arrival timing, Koch and Narum (2020) discovered that the strength of association varied strongly between Columbia River Basin lineages, with up to 9% of phenotypic variance explained in ST Chinook salmon but >70% in OT.

Here, we expand upon previous studies by coupling individual genetic and run timing phenotypes for Chinook salmon that were sampled and PIT tagged early in their return migration to freshwater and then tracked through the Columbia River Basin until they arrived to natal tributaries near their spawning grounds. This study addressed three specific questions regarding the association of *GREB1L* and *ROCK1* with two adult migration phenotypes (freshwater entry and arrival timing) in Chinook salmon: (1) Is the pattern and strength of association the same among the three lineages? (2) How much variation for the two migratory phenotypes is explained by candidate genes (*GREB1L*/*ROCK1*)? and (3) Do the candidate genes demonstrate dominant or additive inheritance patterns for migratory phenotypes? Our characterization of these patterns improves the utility of these candidate genomic regions for management applications in Chinook salmon populations that exhibit variation in adult migration timing.

## 2 METHODS

### 2.1 Samples

All samples were collected with nonlethal methods at the Bonneville Dam Adult Fish Facility (BONAFF) between 2017 and 2019. This facility is located adjacent to a north shore fish ladder of Bonneville Dam and enables a portion of returning fish to be nonlethally sampled during their adult migration. Samples were collected during the entire run from April through October except for when the water temperature exceeding 22.2°C. Total sample sizes were estimated to be <1% of the run (Hess et al., 2021), and higher sampling rates were not possible given limitations of the facility. Metadata was recorded for each corresponding fish sampled including passage date at the facility and fork length, a PIT tag was inserted (unless previously tagged), scales were taken for aging, and a fin clip was taken for genetic analysis. Fish were then released into a recovery pool to volitionally continue their migration. Scales were read to determine ocean duration following standard practice for aging salmonids.

### 2.2 Genotyping

DNA was extracted from fin clips using nondenatured Chelex protocols from the manufacturer (Sigma‐Aldrich). A panel of 343 markers, SNPs, and insertion‐deletion sites (Hess et al., 2021; Janowitz‐Koch et al., 2019) were genotyped using GT‐seq (Campbell et al., 2015). The majority of markers in the SNP panel were putatively neutral, but also included a marker predictive of sex (Brunelli et al., 2008), as well as F_ST_ outliers from landscape analyses (Hecht et al., 2015). Further, the panel included 28 markers in the region of chromosome 28 associated with migration timing, with five in the *GREB1L* gene, eleven from the intergenic region, and twelve from the *ROCK1* gene (Koch & Narum, 2020). All genotype and phenotype data, and R code to process them, have been deposited in the DRYAD repository (https://datadryad.org/stash/share/4t7rmcqThSpoOCn‐YSRHz7jYwvAFxpUhDnE3ubmSt7Y).

**FIGURE 1:**
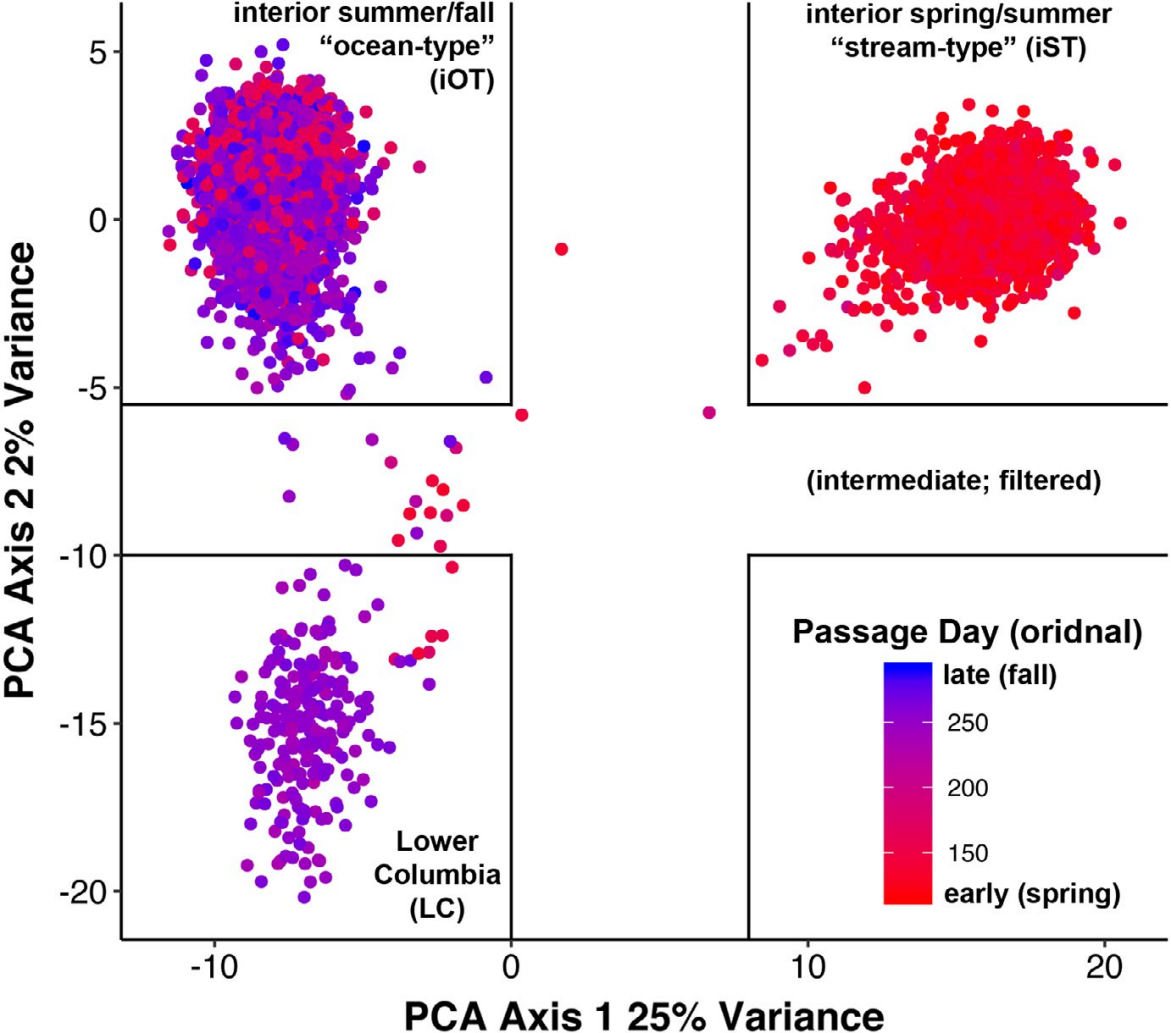
Loadings on the first two principal components from an analysis of putatively neutral loci in Columbia River Basin Chinook salmon. Solid lines demarcate where fish of indeterminate genetic affiliation were omitted. The ordinal date at which each fish were tagged crossing Bonneville Dam is imposed as color on these loadings (red, earlier; blue, later)

Data were filtered to retain samples with <10% missing data and formatted using custom code in R (R Corp.) and pgdspider (Lischer & Excoffier, 2012). Putative neutral loci (265) were used to identify individuals to each of the three lineages (LC, IOT, and IST) based on a principal components analysis performed using adegenet v2 (Jombart, 2008) in R; individuals that exhibited ambiguous lineage affinity were omitted (Figure 1). For each lineage separately, this neutral dataset was filtered for linkage disequilibrium and effects of selection (outlier loci) using the exact test implemented in genepop v1.1.7 (Raymond & Rousset, 1995), with 50 batches of 50 iterations after 50 of dememorization, and outflank v0.2 (Whitlock & Lotterhos, 2015), with default options except for a RightTrimFraction of 0.1, respectively. Loci with *q*‐values <0.1 were omitted, producing a “neutral and unlinked” marker set for each lineage. While different programs for outlier identification vary in their efficacy across demographic scenarios and genetic data types (Hoban et al., 2016), by using liberal thresholds with this relatively heuristic method, our “neutral” dataset could be considered conservative with respect to the potential effects of selection and was congruent with previous studies of neutral variation (Koch & Narum, 2020). We examined linkage disequilibrium in the candidate region of chromosomes 28 for each lineage using the program haploview v4.2 (Barrett et al., 2005). For simplicity hereafter, we refer to the SNP markers in the candidate region of chromosomes 28 by their order of sequence along the chromosome: 1 through 28, respectively. These numbers differ slightly than those used in Koch and Narum (2020) since five markers were dropped due to inconsistent or poor amplification (see Table S1).

### 2.3 Migration timing phenotypes

**FIGURE 2:**
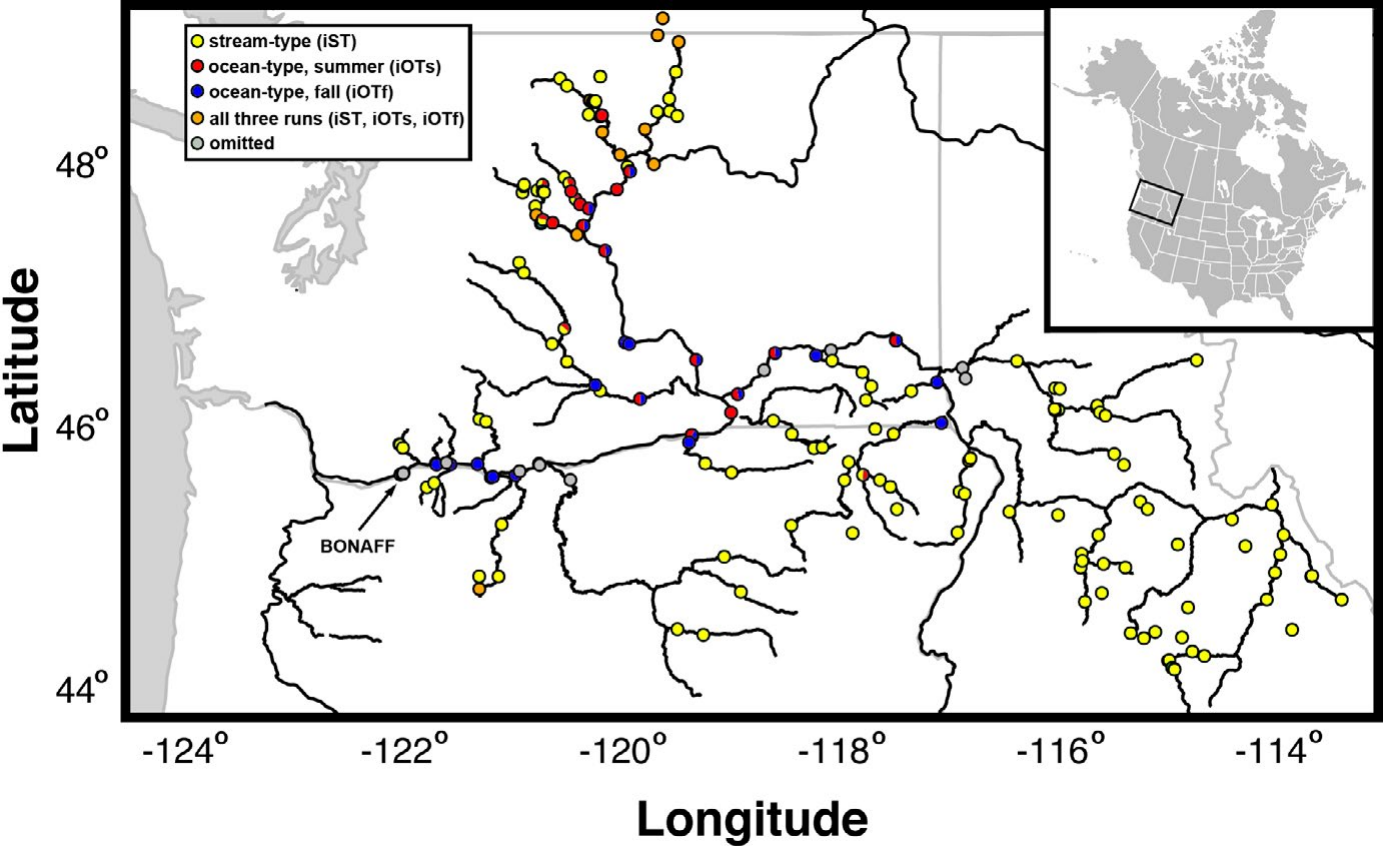
Locations of passive integrated transponder (PIT) arrays in the Columbia River Basin where Chinook salmon tagged at Bonneville Dam were later recorded for the last time. Active arrays for each lineage separately are included as Figure S1. Lower Columbia lineage fish were only recorded at Bonneville Dam, identified as the farthest west gray array

For all Chinook salmon sampled at Bonneville between 2017 and 2019 (*N* = 8327), we obtained complete PIT histories from PTAGIS (https://www.ptagis.org). These were filtered using custom code in R, as follows. Chinook salmon migrating to the interior Columbia R. encounter Bonneville Dam at river kilometer 234, and the date of this arrival (“Passage Day”) serves as a common reference point for fish passage (Figure 1), a date that may be proportional to freshwater entry. Subsequently, the most upstream array at which each fish was recorded was expected to be the closest to the tributary in which they spawned, which approximates arrival date (“Arrival Day”). Mortalities that occurred due to harvest, predation, or other unknown causes exhibited incomplete migration histories, providing abbreviated estimates of arrival date. These were recognized when an individual was last recorded at a site where spawning was not expected, and these samples (~3k) were omitted. Because fish in different lineages and runs tend to use different reaches for spawning, the arrays from which records were omitted were different. Recordings at arrays lower in the basin were omitted for iST Chinook salmon but retained for iOT, with the exception of some middle Columbia river mouths, which were omitted for summer‐run iOT Chinook salmon (Figure 2 and Figure S1). Although some prespawn mortalities were likely included in the final dataset, being relatively conservative in which arrays were allowed ensured that predicted Arrival days could be off by only a small amount. Passage Day was used to discriminate summer and fall run within the iOT lineage, based on the observed minimum in passage (July 31, ordinal day 212), which permitted identifying incomplete migration histories for summer‐run iOT fish. Some LC lineage fish spawn in east of the Cascades in the reach above Bonneville Dam and the lower reaches of nearby tributaries (i.e., Spring Creek). The last PIT array these fish pass before spawning will often be at Bonneville Dam, making Passage and Arrival Days the same for most LC lineage fish. For this reason, we did not utilize Arrival Day for LC Lineage fish. PIT arrays were also grouped by subbasin (Figure S2) to calculate two relative statistic pairs: each fish's Passage and Arrival day was transformed relative to the (1) median or (2) last Passage or Arrival day, respectively, for all arrays grouped by subbasin. This gave us a total of six run timing statistics: raw (ordinal) Passage and Arrival Days, and Passage and Arrival Days relative to the median or last such day for each subbasin. Finally, we transformed Passage or Arrival Days into categorical variables, as follows: For iOT fish, we coded Passage Day as a binary variable that described passage on or before versus after ordinal day 212 (July 31, the last day of the summer management period). Chinook salmon management periods (defined in U.S. vs. OR Management Agreement) pertain to passage days at Bonneville Dam and include the Spring (January 1–June 15), Summer (June 16–July 31), and Fall (August 1–December 31) Periods. For iST fish from six subbasins with sufficient samples (*N* ≥ 27; Salmon, Clearwater, Grande Ronde, Deschutes, Yakima, and Wenatchee), we visually identified minima in Arrival Day density plots for each subbasin (Figure S3) and categorized each fish in these subbasins as arriving before or after this day.

### 2.4 Association testing

We tested the association of all 342 autosomal SNPs with run timing in each lineage using GAPIT v. 20190926 (Lipka et al., 2012). We used the 200 (ST), 223 (OT), and 265 (LC) “neutral and unlinked” loci to infer a kinship matrix and three principal components reflecting underlying population structure in each lineage. Fork length (mm), ocean duration (years), sex (as 1 or −1), and hatchery origin (0 or 1) were included as covariates. Missing values in length and ocean duration were imputed as the median value for that lineage, while missing sex was coded as zero (missing covariates <10%). The putative genetic stock and hatchery origin of each fish were estimated elsewhere using parentage‐based tagging and genetic stock assignment (Hess et al., 2018, 2019, 2020). We ran the “mixed linear model” (MLM) of GAPIT. To filter SNPs that are in perfect linkage disequilibrium in each lineage, we estimated LD among all SNPs using PLINK v1.9 (Purcell et al., 2007) and retained only a single representative from each LD grouping with *r* ^2^ >0.99. The net proportion of phenotypic variance explained by each SNP ($R_{\text{SNP}}^{2}$) from these MLM models was calculated as $R_{\text{SNP + covariates}}^{2}$ − $R_{\text{covariates - only}}^{2}$. To test the independence of effects on Arrival Day from Passage Day in iST and iOT Chinook salmon, we ran a model for Arrival Day with Passage Day as a covariate, as well models with the residuals of regressing ordinal Arrival Day and relative‐subbasin Arrival Days as the phenotypes (linear regression was made using the *lm* function, stats package in R). Finally, we tested the potential for a dominant effect of chromosome 28 variation on ordinal Passage and Arrival Days by coding heterozygotes as either homozygote in turn. While not a formal test, stronger associations of either two‐genotype coding over three genotypes, as measured by the proportion of phenotypic variance explained ($R_{\text{SNP}}^{2}$), would suggest a dominant effect of one allele over the other (Balding, 2006).

For ordinal Passage and Arrival Days, we also ran the “Bayesian information and Linkage disequilibrium Iteratively Nested Keyway” (BLINK) model in GAPIT, which groups loci according to linkage disequilibrium (LD) thresholds and tests a representative SNP from each LD group, orders these by association, and then tests the significance of subsequent LD groups while including higher ranked LD groups as covariates. We modified the BLINK code to group only loci in near‐perfect LD (≥0.99), implying that differences in significance between the MLM and BLINK models reflect the redundancy in explanatory power produced by the remaining linkage among loci. The BLINK model does not report the variance explained by each LD group (*R* ^2^), so for the first 12 LD groups reported by BLINK, we used sequential MLM models in which cluster‐representative SNPs were included as covariates to estimate *R* ^2^. Missing covariate genotypes in these sequential MLM models were input as heterozygotes.

Since SNPs in LD were not independent, further examination was made through haplotype association in the candidate regions. However, as results from haplotype‐based analyses were strongly consistent with the SNP‐based methods, we describe these analyses and results in the supplementary information archived with DRYAD.

## 3 RESULTS

**FIGURE 3:**
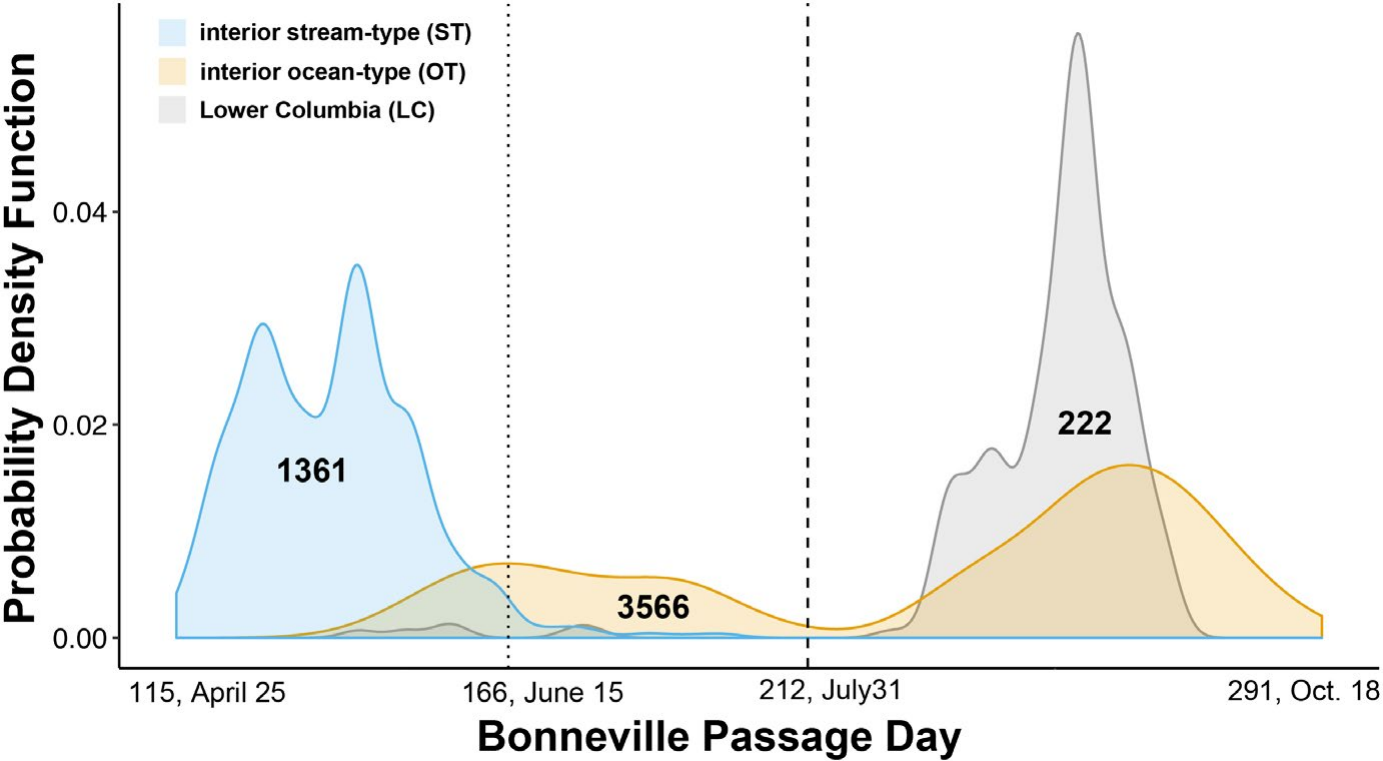
Probability Density Function for the date at which each Columbia River Basin Chinook salmon was tagged crossing Bonneville Dam between April and October, for each lineage (blue, interior stream‐type; orange, interior ocean‐type; gray, Lower Columbia). Probability Density Function is akin to the proportion of fish in a lineage that passed on each day. For each color, the area under the curve sums to 1, but curves are not proportional to each other in counts; the sample size representative of each curve is indicated. Vertical dotted and dashed lines represent divisions between the spring, summer, and fall management periods, ordinal, and calendar dates for which are noted, along with the first and last day a Chinook salmon in this dataset migrated

A total of 5149 Chinook salmon migrated to the arrays identified for each lineage (Figure 1): 222 from the LC lineage, 3566 from the interior ocean‐type (iOT) lineage (1234 summer run and 2332 fall run), and 1361 from the interior stream‐type (iST) lineage (Figure 2 and Figure S1). Genotyping was repeated as necessary so that samples had >90% or greater genotype completeness. The filtered dataset was representative of adult and jack‐sized Chinook salmon including clipped and unclipped hatchery‐origin and unclipped natural‐origin collected in the BONAFF that include all three management periods in this region (Spring *N *= 1794, Summer *N* = 804, and Fall *N* = 2551). Genetic stock analysis identified most of the Spring Period fish to be predominately iST lineage from Snake River tributaries (*N* = 718, ~40%), a third were ST lineage from mid and upper Columbia spring‐run stocks (*N* = 597), and a quarter were iOT lineage from the upper Columbia summer run (*N* = 451). No more than 1% (*N* = 20) were from the LC lineage spring‐run stocks. The Summer Period fish were comprised mostly of iOT fish from the upper Columbia River summer‐run stock (*N* = 756, 94%), approximately 3% were from the iST lineage from middle and upper Columbia River and Snake River spring‐run stocks (*N* = 27), and less than 1% were LC lineage spring‐run stocks (*N* = 3). Finally, the Fall Period fish were 91% from the iOT lineage destined for middle and upper Columbia River and Snake River reaches (*N* = 2330), and the remaining 9% were of the LC lineage (*N* = 221) predominated by hatchery‐origin Spring Creek Hatchery tules (*N* = 211, Table S2). We omitted 20 individuals that exhibited ambiguous lineage affinity; these fish were distributed across all three management periods, but were mostly comprised of LC lineage fish (*N* = 16), many of which were clipped hatchery‐origin fish from the Willamette River spring‐run stock (Table S2). We have described straying above Bonneville Dam from this LC stock previously (Hess et al., 2014). As expected given the stock composition, the iST Chinook salmon all arrived early in the year, but overlapping with “early” or summer‐run iOT fish (Figure 3). While a few LC Chinook salmon arrived during the summer‐run period (*N* = 6), the majority of those migrating east of the Cascades crossed Bonneville Dam with the fall run after ordinal day 212.

**FIGURE 4:**
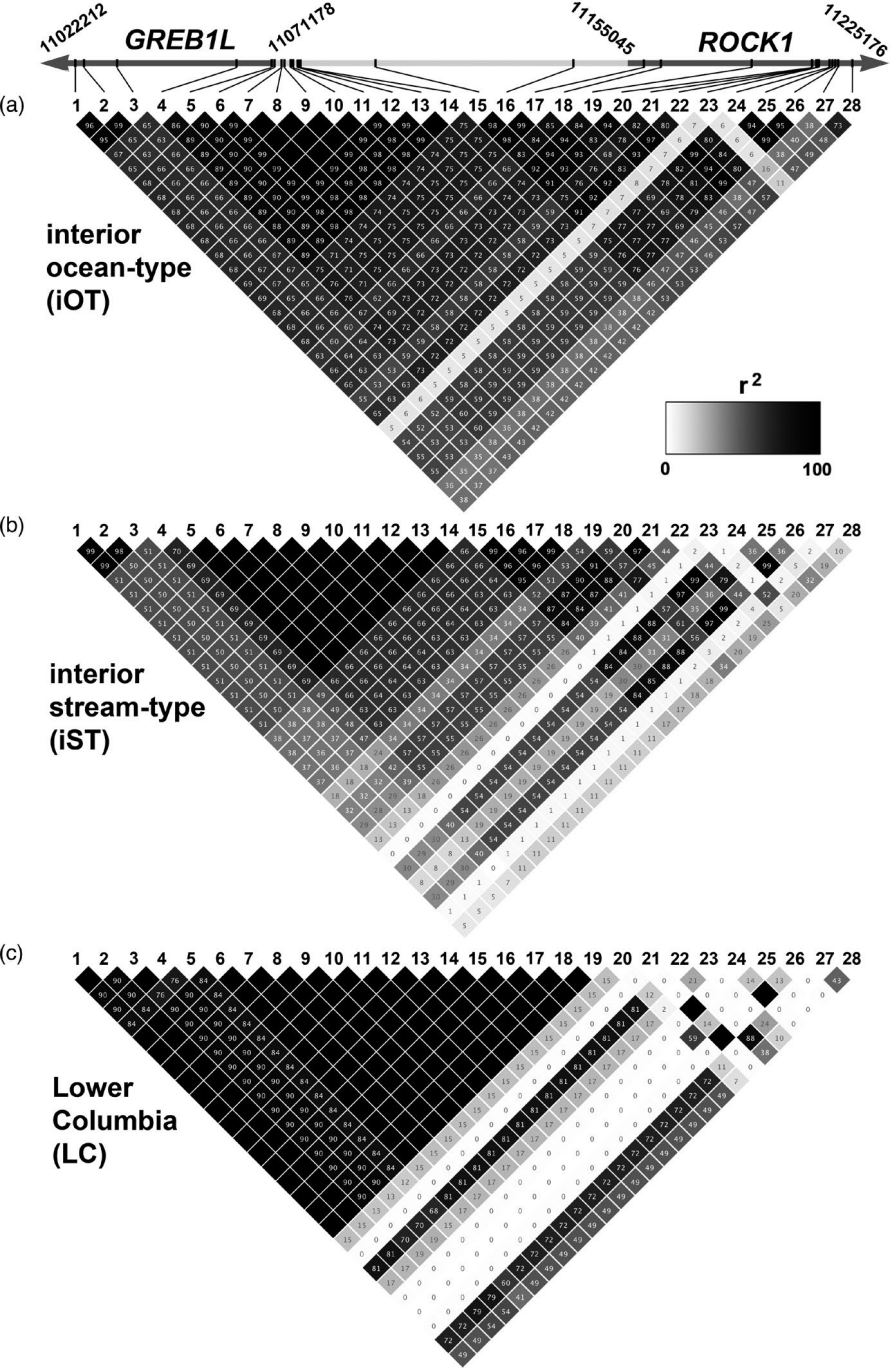
Linkage (*r* ^2^ values) for chromosome 28 candidate markers in Chinook Salmon, and relative spacing of those markers, with shading on the positional reference indicating the genic or intergenic regions, and arrow indicating the direction of transcription of the two genes. The numbers indicate the genomic positions of the beginning and end of each gene. Top: interior ocean‐type; middle: interior stream‐type; bottom: Lower Columbia

Patterns of linkage within the chromosome 28 candidate region were similar among lineages (Figure 4), with markers in the center of the region (the upstream portion of GREB1L, intergenic, and upstream portion of ROCK1; approximately markers 4–20) showing stronger LD than those on either periphery. Within this region, there were two subgroups with *r* ^2^ between 0.95 and 1 for iST and iOT fish containing markers 5 to 14 and 15 to 18, although the endpoints of the left‐central (*GREB1L*‐side) and right‐central (*ROCK1*‐side) LD blocks varied slightly between iST and iOT fish (5 vs. 6 and 17 vs. 18). LC fish exhibited a single central, high LD block that extended from marker 6 through marker 19.

**FIGURE 5:**
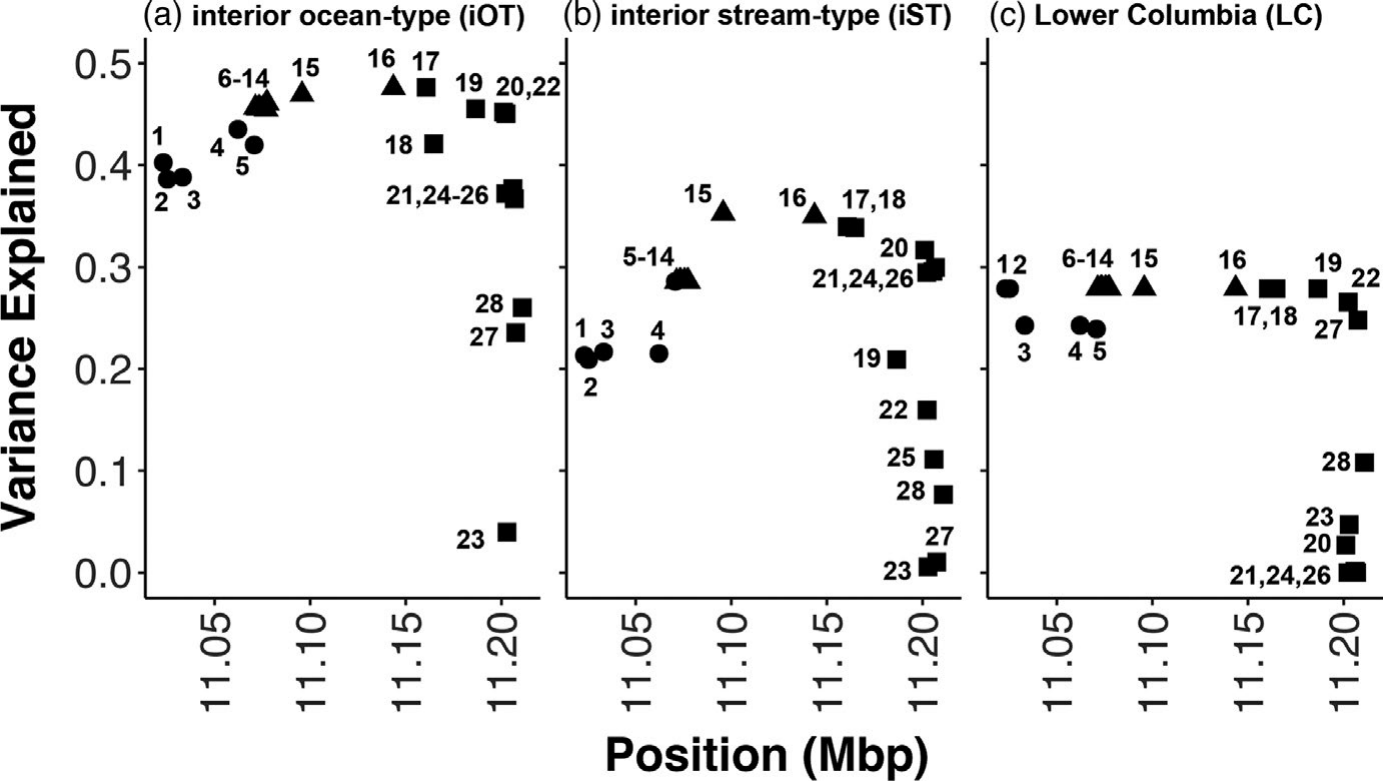
Strength of association (*R* ^2^) for Passage Day by genomic position in the chromosome 28 candidate region of Chinook Salmon, where shape indicates genomic structure (circle: GREB1L, triangle: intergenic, square: ROCK1) and the number represents the marker order (positions provided in Table S1). The *x*‐axis represents chromosome position in millions of base pairs (Mbp). Left: interior ocean‐type; middle: interior stream‐type; right: Lower Columbia

The dates that fish with different genotypes in the chromosome 28 candidate region crossed Bonneville Dam (Passage Day) and were recorded at their most upstream array (Arrival Day) showed a distinct pattern (Figures S4, –S8). Association tests in GAPIT confirmed that the SNPs in the candidate region of chromosome 28 stood out from all other SNPs in strong association with run timing variables (Figure S9 and Files [Link], [Link], [Link]). In iOT and iST fish, markers in the right‐central linkage block (*ROCK1*‐side) showed the strongest association with both ordinal Passage Day and Arrival Day, followed by markers in the left‐central block (*GREB1L*‐side; Figure 5 and Figure S10). For LC lineage fish, there was no distinction among SNPs in the single central LD block (markers 6–19), which showed the strongest association (Figure 5). However, the maximum variance in Passage Day explained by these SNPs was different in each lineage, with iOT Chinook salmon showing the strongest association (max *R* ^2^ = 0.476), followed by iST Chinook salmon (*R* ^2^ = 0.353) and LC (*R* ^2^ = 0.279), though we note the small sample size of spring‐run LC Chinook salmon compromises the precision of the variance estimate for that lineage (Files [Link], [Link], [Link]). The maximum variance explained in Arrival Day was also higher in iOT (*R* ^2^ = 0.396) than iST fish (*R* ^2^ = 0.098), and, notably, each was less than the respective values for Passage Day, though this discrepancy was larger for iST fish. The pattern and amount of variance explained in the subbasin relative variables showed similar patterns and in each case were less than the raw ordinal variables (Files S1,S2). Results from the BLINK analyses of ordinal Passage and Arrival Days reflected these results, with the group containing the right‐central LD block (*ROCK1*‐side) identified as the most significant, while all other LD groups explained a much smaller amount of variance in run timing after accounting for the earlier LD groups (Figure S11). In most cases, the second most significant LD group was also in the chromosome 28 candidate region. Association testing with haplotypes estimated from genotypes of the chromosome 28 candidate markers also substantiated these observations (results archived on DRYAD).

**FIGURE 6:**
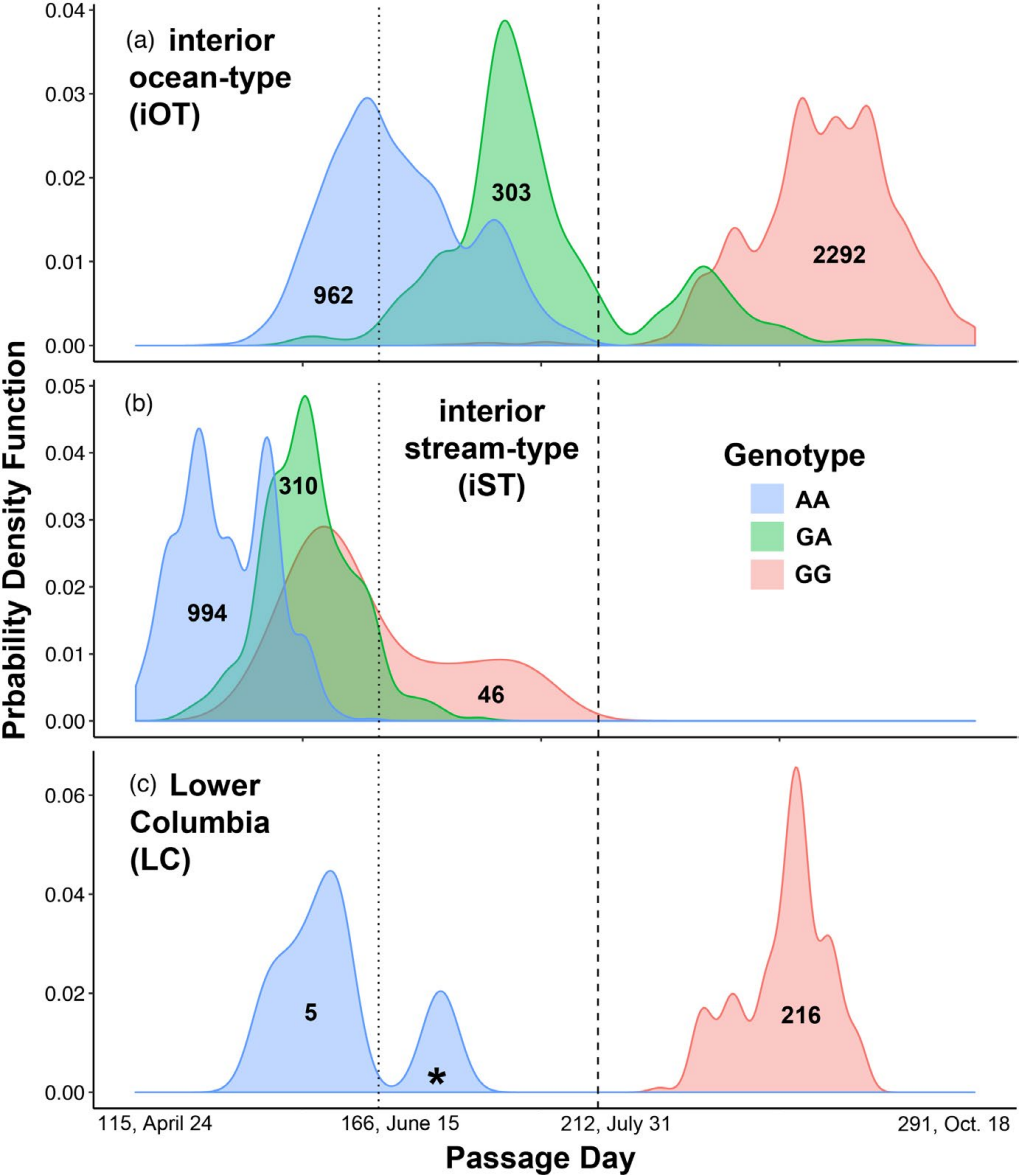
Probability Density Function for Passage Day by genotype of Chinook Salmon for a representative marker (marker 16, Ots28_11143508) from the most strongly associated linkage group. (a) interior ocean‐type; (b) interior stream‐type; (c) Lower Columbia (the passage of a single heterozygote is indicated with an asterisk). Probability Density Function is akin to the proportion of fish in a lineage with a given genotype that passed on each day. For each color on each panel, the area under the curve sums to 1, but curves are not proportional to each other in counts; the sample size for each curve is indicated. Vertical dotted and dashed lines represent divisions between the spring, summer, and fall management periods, ordinal and calendar dates for which are noted, along with the first and last day a Chinook salmon in this dataset migrated

The evidence for dominance effects in chromosome 28 variation differed by lineage, although for the SNPs in the most strongly associated linkage groups identified above, heterozygotes were generally intermediate in Passage Day to homozygotes, both overall (Figure 6) and within individual subbasins with sufficient sampling of each genotype (Figure S12). For iST Chinook salmon, coding heterozygotes so that they were either of the two homozygotes did not change the patterns of run timing association within the chromosome 28 region, and neither of these dominance codings explained as much variation as codominance, although the strength of association was stronger when coding heterozygotes as early‐migrating homozygotes than later migrating (File S1). LC lineage Chinook salmon showed a similar pattern of association across all three heterozygote codings, but the strength of association was slightly higher than codominant when heterozygotes were coded as early‐migrating (premature) homozygotes (File S3). For iOT Chinook salmon, neither coding for heterozygotes explained as much variance in Passage or Arrival Days as codominance, although the strength of association was stronger when coding heterozygotes as early‐migrating homozygotes than later migrating (File S2). However, the pattern of association within the chromosome 28 candidate region changed also, with the left‐central LD block (*GREB1L*‐side) showing a stronger association than the right‐central LD block (*ROCK1*‐side) when heterozygotes were coded as the late‐migrating (mature) homozygote, though these values were all still smaller than the default codominant coding. Interestingly, when iOT Chinook salmon Passage Day was coded as categorical (before or after July 31; ordinal 212), the pattern of association was marginally stronger for the left‐central LD block (*GREB1L*‐side) rather than the right (File S2). In contrast, when the Arrival Day of iST Chinook salmon from six subbasins was coded categorically (before or after a subbasin specific day), there were no significant associations with chromosome 28 (File S1). Perhaps relatedly, when Passage Day was used as a covariate in the MLM model, or when residuals from regression of Arrival Day variables on Passage Day were tested, there was no longer an association with chromosome 28 variation in either the iOT or iST lineages, suggesting that the effect of chromosome 28 variation on Arrival Day is not independent of that on Passage Day (Files S1,S2).

## 4 DISCUSSION

Recent studies have shown that the *GREB1L*/*ROCK1* region on chromosome 28 has a strong effect on run timing in Chinook salmon and steelhead trout (*Oncorhynchus mykiss*), but many questions remain about the consistency and strength of this genetic variation among the various populations of each species and thus the utility of identified molecular markers from this region (Waples & Lindley, 2018). Our results confirm that genetic variations in this region are associated with effects in the same direction in each of the three lineages of Chinook salmon present in the Columbia River Basin, albeit in a lineage‐specific strength and pattern, and thus that genetic markers in this region will be useful in predicting run timing distributions of stocks in the Columbia Basin. Moreover, heterozygotes at the most strongly associated markers were largely intermediate in their migration timing relative to homozygotes, suggesting that strong selection for/against a particular run will largely preclude the retention of variation for alternative migration phenotypes.

We observed that markers in the chromosome 28 region explained a large portion of phenotypic variation in the date around which Chinook salmon began their migration (Passage Day), and to a lesser extent, the approximate date on which they arrived to their spawning grounds (Arrival Day). Consistent with the linkage among these markers and the strong haplotype blocks in the central portion of this genomic region, the same haplotypes (combination of SNP alleles) were strongly associated with run timing variation in each of the three lineages. This provides strong evidence that the same genetic variation has similar effects in each of the three lineages, albeit in a lineage‐specific manner. For example, although Lower Columbia (LC) and interior ocean‐type (iOT) Chinook salmon appear to exhibit similarly large differences in run timing phenotypes, both presenting a larger range between early and late migrators than interior stream‐type (iST), the strength of association between chromosome 28 alleles was considerably higher for iOT Chinook salmon than for LC (max *R* ^2^ = 0.476 vs. 0.279, respectively). While this difference could conceivably result from strong asymmetry in representation of the two runs in our LC samples (*N* = 6 vs. *N* = 216), a previous study (Koch & Narum, 2020) observed a similar effect size (max *R* ^2^ = 0.280) but with symmetrically distributed collections of Chinook salmon from the Cowlitz tributary of the lower Columbia (*N* = 94 each for early (spring) vs. late (fall) collections). In contrast, this same previous study also observed considerably higher association values for iOT Chinook salmon collected at Prosser and Wells Dams (max *R* ^2^ = 0.71), suggesting that the estimates made herein may yet be conservative. Two differences between these two studies should be noted. First, while the current study considered iOT Chinook salmon from a number of different interior spawning groups (tributaries and reaches), those authors used early‐ and late‐migrating collections from within a single representative population of each lineage (Koch & Narum, 2020), thus avoiding some potential confounding variation that we may not have been able to completely accommodate through covariates here. Second, while we considered individual run timing data (i.e., continuous response variable) gleaned from PIT tag recordings and passage date, this previous study used a categorical (binary) response variable based on the designation of each population as early or late arrival for spawning. However, we did not see strong differences in results after transforming our migration data into a categorical response, except for changing rank among markers in the left‐central (*GREB1L*‐side) and right‐central (*ROCK1*‐side) LD blocks, suggesting this coding is not responsible for the difference in the magnitude of association between the two studies.

The relative strength of the association of run timing of Columbia Basin Chinook salmon with markers in the intergenic region between the *GREB1L* and *ROCK1* genes is notable for its consistency both here and in Koch and Narum (2020), as well as similar studies of spring vs. fall Chinook salmon from coastal populations (Thompson et al., 2019; Thompson et al., 2020). While the strongest associations appeared to be with markers linked to the most upstream portion of the *ROCK1* gene both here and in Koch and Narum (2020), the markers in the linkage group including the most upstream portion of *GREB1L* showed values of nearly equal magnitude, while markers adjacent but further downstream in the *ROCK1* gene showed considerably weaker associations. Interestingly, Columbia Basin steelhead, which show a similar association of chromosome 28 variation as Chinook salmon, in some cases explaining as much as 50% of variation in run timing, exhibited the strongest associations with the upstream portion of *GREB1L* and adjacent intergenic region (Willis et al., 2020). This slight difference in genomic localization of the strongest signal of association across these two species is unexpected because parsimony would suggest similar effects of chromosome 28 genetic variation on these two species (Ford et al., 2020). However, it is worth noting that, despite the fact that these fishes have so far been the only salmonid species in which chromosome 28 variation has been implicated in run timing, there are reasons to expect that patterns of association would differ between them, including that they are not each other's closest relatives. Moreover, as these two genes are situated with opposing transcriptional frames, they may share a regulatory region, and so their effects on run timing may not be independent (Narum et al., 2018). While both genes are expressed in reproductive and renal tissues and appear to be integral to development in humans (Brophy et al., 2017; De Tomasi et al., 2017; Mizuno et al., 2013; Nakagawa et al., 1996; Oviedo et al., 2011; Sanna‐Cherchi et al., 2017), their functions in fishes are still uncertain. Thus, while markers in this chromosome 28 region appear to have strong predictive value for run timing in Chinook salmon, studies illuminating the functional structure of the intergenic sequence in this region and the effects of observed genetic variation would be very useful.

Although interior stream‐type (iST) Chinook salmon showed a similar pattern of association as the other lineages with strongest associations of markers 15–17, in the intergenic region, and similar association signals for similar haplotypes, which is notable considering how divergent this lineage is from the other two, there were two additional noteworthy observations specific to iST Chinook salmon. The first is that the magnitude of association between individual run timing and chromosome 28 variation was similar between the three lineages despite earlier median run date and more constrained overall variation in run timing in iST Chinook salmon compared to iOT or LC, and all iST Chinook salmon are reproductively immature at the time of freshwater entry (Myers et al., 1998). This suggests that although chromosome 28 alleles appear to have very similar effects, some other aspects of genomic background in this lineage act to further modify those effects. Second, the magnitude of association with individual run timing for iST Chinook salmon observed here was considerably larger for Passage Day (max *R* ^2^ = 0.353) than Arrival Day (*R* ^2^ = 0.098), but estimates of association for Arrival Day between the current study and Koch and Narum (2020) were very similar. On this point, our results for iST and iOT Chinook salmon provide further understanding regarding phenotypic association, since the difference in signal of association between Passage Day and Arrival Day was less for iOT (difference of 0.080 in *R* ^2^) than for iST fish (difference of 0.255 in *R* ^2^). Thus, it appears that the main difference between these studies in this result is the characteristic of run timing phenotypes that were tested. Nonetheless, the difference in the degree to which Arrival Day mirrors Passage Day in these two lineages is interesting, considering that analyses factoring out the correlation of Arrival Day with Passage Day indicated that the effects of chromosome 28 variation on these days are not independent, and despite the possibility that some discrepancy in run timing may occur between actual freshwater entry and passage at Bonneville Dam, 234 km upstream of the Columbia River estuary. This observation suggests that more diverse or extreme barriers or environmental variation along the migration paths of iST Chinook salmon act to diminish the correlation between freshwater entry and arrival to spawning grounds relative to iOT fish (Hecht et al., 2015).

While spring and summer‐run Chinook salmon are at an advantage over later returning fish by utilizing habitats that might be inaccessible later in the year, there appear to be tradeoffs associated with an early run timing strategy (Quinn et al., 2016). In particular, later‐migrating fish avoid the highs in river temperature during the summer that make migration physiologically more taxing (Quinn et al., 2016), and given the more extreme temperatures and variation in flows associated with climate change, and it is not surprising that early‐migrating (spring and summer run) Chinook salmon have been beleaguered disproportionately to later‐migrating (fall or winter run) fish, even so far as being extirpated completely in some cases despite the persistence of strong fall runs (Ford et al., 2020; Thompson et al., 2019). An important uncertainty is if the conditions for early‐migrating fish were to improve where such a run once existed, could early‐migrating runs re‐emerge from nearby later‐migrating fish of the same lineage (Ford et al., 2020; Koch & Narum, 2020; Thompson et al., 2019; Waples & Lindley, 2018)? Since we now recognize the importance of chromosome 28 variation in shaping migration phenotypes, the answer to this often depends on the migration phenotype of heterozygotes carrying the early migration (premature) allele, and the degree to which these persist in the fall run (Ford et al., 2020; Koch & Narum, 2020; Thompson et al., 2019; Waples & Lindley, 2018). Examining phenotype plots of the most significantly associated marker with run timing, heterozygotes in iOT and iST fish appear to migrate at an intermediate time to either homozygote, suggesting the premature allele is not recessive and will not persist cryptically within later‐migrating runs. We note that slightly more heterozygote iOT fish migrated with the peak of homozygous early/premature than with the peak of homozygous late/mature fish, but this may be a product of low heterozygote fitness during the period of highest temperatures rather than direct genetic effects (Narum et al., 2018). While our examination of patterns of inheritance of the chromosome 28 alleles did not reveal clear evidence for dominance, there were indications of partial dominance of the early migrating (premature) rather than late‐migrating (mature) alleles, further eroding the hope that genetic variation for premature migration could be retained within later‐migrating runs.

Finally, these genetic markers have the potential to be useful for tracking and predicting migration phenotypes for closely managed stocks of Chinook salmon. Chinook salmon in the Columbia River mainstem are managed by ESA‐listed stocks that are mostly contained within management periods (Spring, Summer, and Fall; Myers et al., 1998). For example, the ESA‐listed upper Columbia River spring‐run and Snake River spring‐run stocks pass Bonneville Dam mostly in the Spring Period, whereas the Summer Period consists largely of a non‐ESA‐listed summer‐run stock from the upper Columbia (National Marine Fisheries Service, 2017). Mainstem harvest rates are higher in the Summer Period relative to the Spring Period to effectively exploit the non‐ESA‐listed stock, but late‐arriving ESA‐listed stocks in the Summer Period can be exposed to nonoptimal harvest rates and there may be missed harvest opportunity when the non‐ESA‐listed stock arrives early in the Spring Period (National Marine Fisheries Service, 2017). Chromosome 28 markers associated with Passage day at Bonneville Dam are variable within these stocks and may prove effective at predicting how much overlap these various stocks will have across the Spring and Summer Periods based on the chromosome 28 genotypes of broodstock fish that were crossed in a hatchery setting. This would provide further information to assist with fisheries management in the region that is heavily based on run timing.

## 5 CONCLUSIONS

Despite strong genomic divergence among lineages of Chinook salmon, genetic variation in the region of chromosome 28 containing the *GREB1L* and *ROCK1* genes was found to have moderate to strong association and similar effects on adult migration timing in each of the three lineages that inhabit the Columbia River Basin. These results point to the potential for these genetic markers to be useful for tracking and predicting migration phenotypes for managed stocks of Chinook salmon, though we recommend that their adoption be made with robust adaptive implementation. Our results align with previous inferences that chromosome 28 variation is codominant, or perhaps marginal partial dominance of the premature allele, suggesting that fall runs of Chinook salmon will be poor long‐term reservoirs and ineffective sources of re‐emergence for premature migration alleles and extirpated spring and summer runs.

## CONFLICT OF INTEREST

The authors declare they have no conflict of interest regarding the data or inferences discussed in this manuscript.

## Supporting information

Fig S1

Fig S2

Fig S3

Fig S4

Fig S5

Fig S6

Fig S7

Fig S8

Fig S9

Fig S10

Fig S11

Fig S12

Table S1

Table S2

File S1

File S2

File S3

## Data Availability

Data, computer code, and supplemental methods and results for this study are available at with DRYAD (https://datadryad.org/stash/share/4t7rmcqThSpoOCn‐YSRHz7jYwvAFxpUhDnE3ubmSt7Y).
