# Supplementary figures and images for: Genomic region associated with run timing has similar haplotypes and phenotypic effects across three lineages of Chinook salmon
Source: Evol Appl. 2021 Sep 1;14(9):2273–85. doi: 10.1111/eva.13290 (PMC8477596; doi:10.1111/eva.13290)

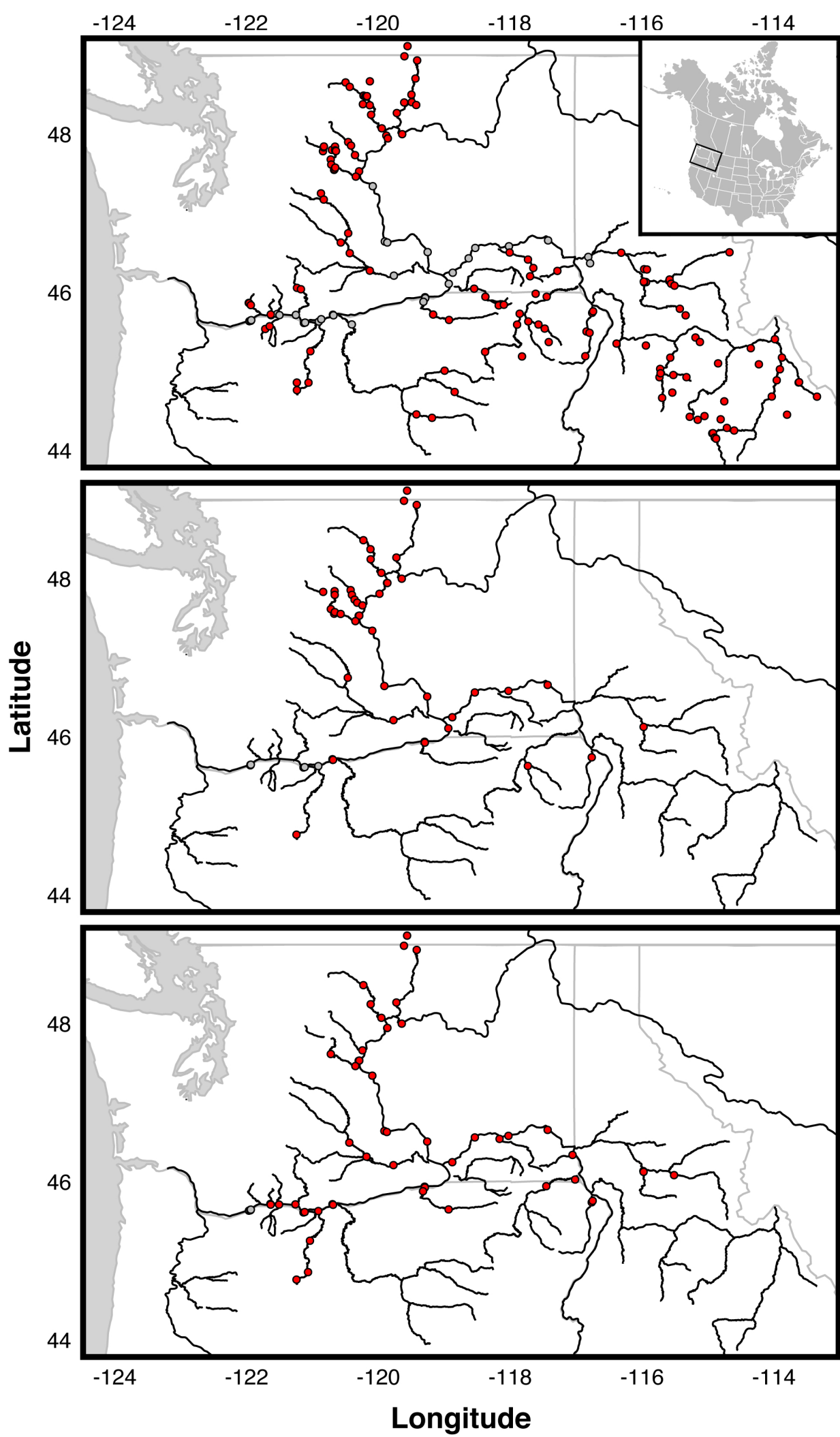

Supplement: Supplementary file 1 — Fig S1 [file EVA-14-2273-s014.pdf]

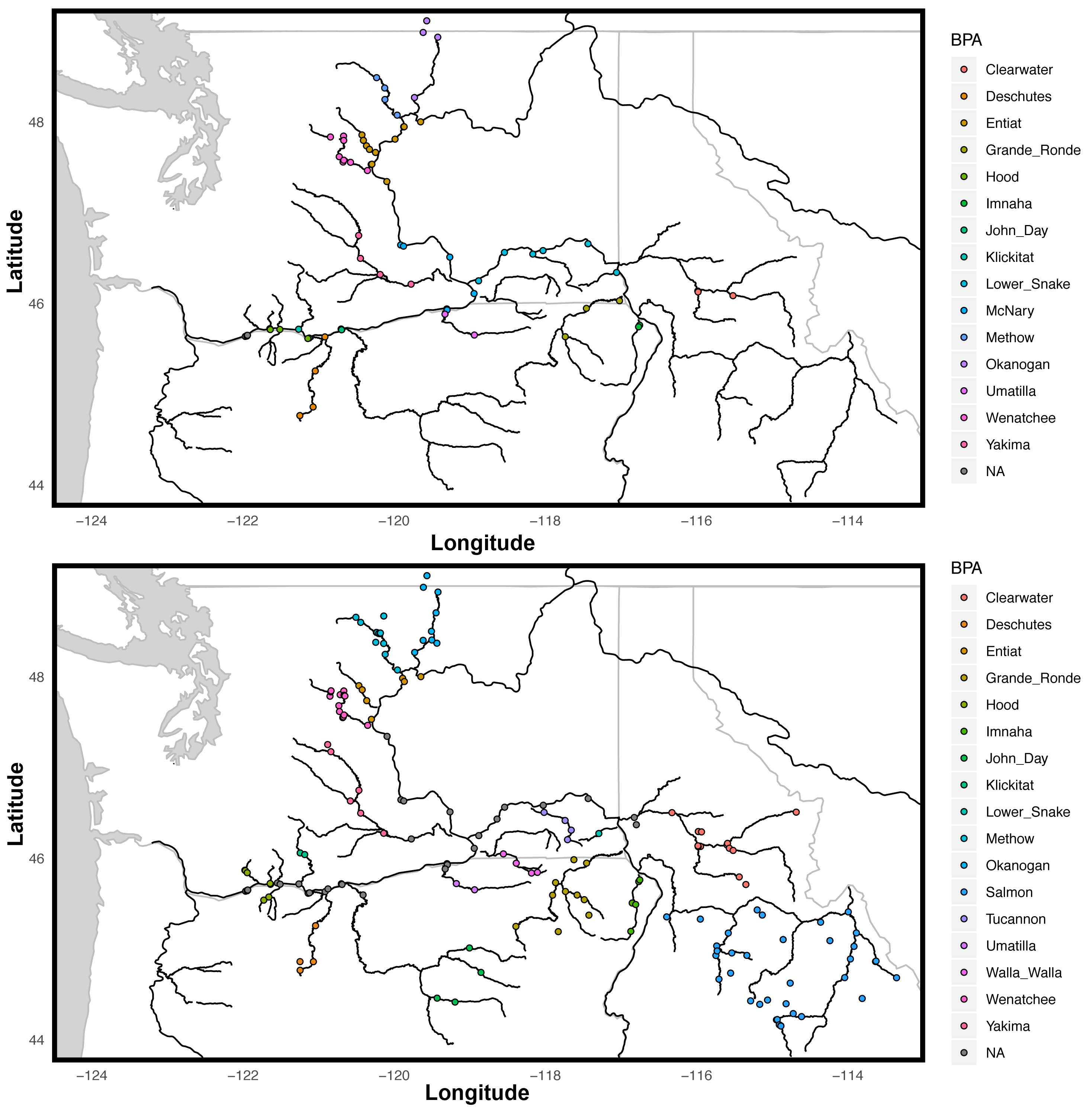

Supplement: Supplementary file 2 — Fig S2 [file EVA-14-2273-s006.tif]

Passage Day

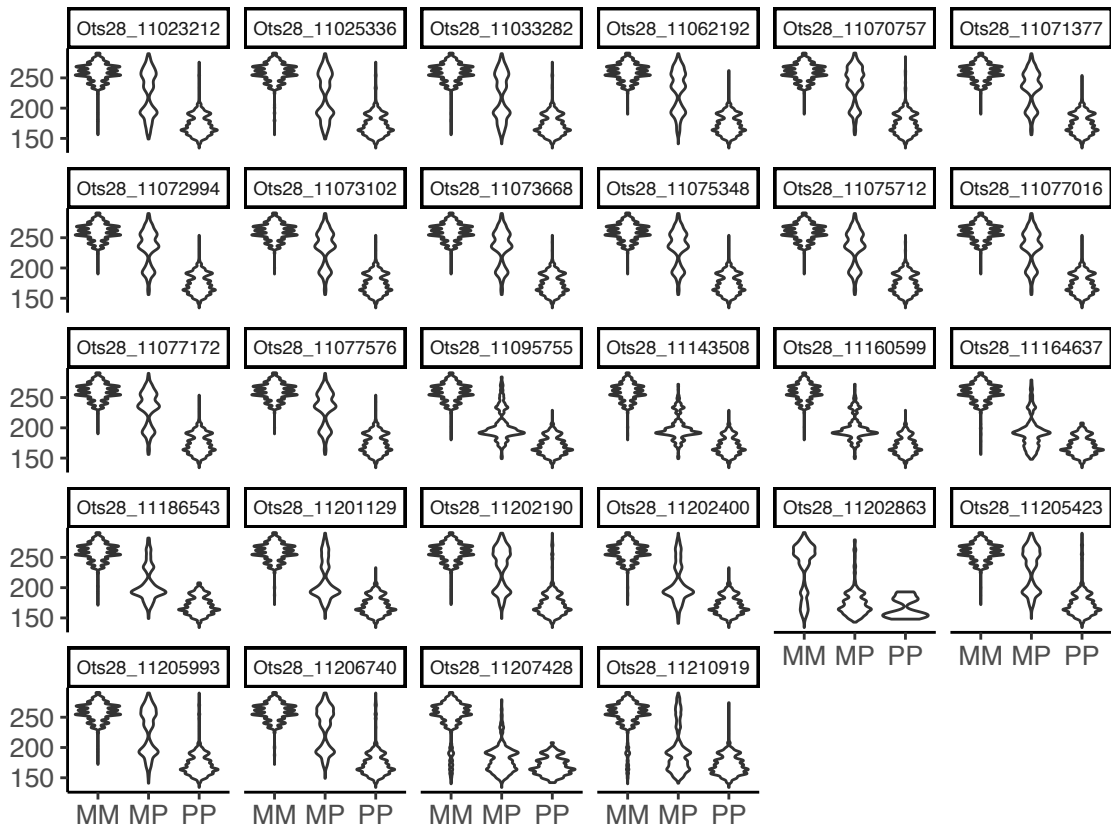

Genotype

Supplement: Supplementary file 4 — Fig S4 [file EVA-14-2273-s001.pdf]

Passage Day

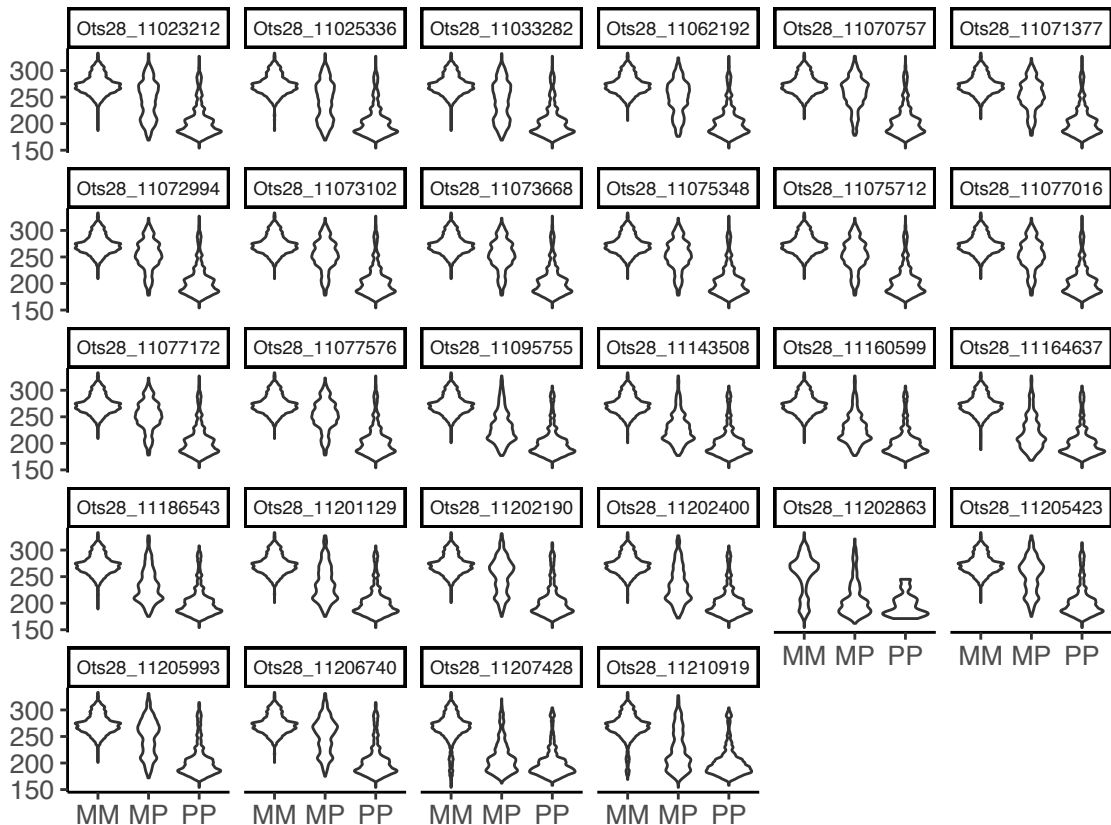

Genotype

Supplement: Supplementary file 5 — Fig S5 [file EVA-14-2273-s007.pdf]

Passage Day

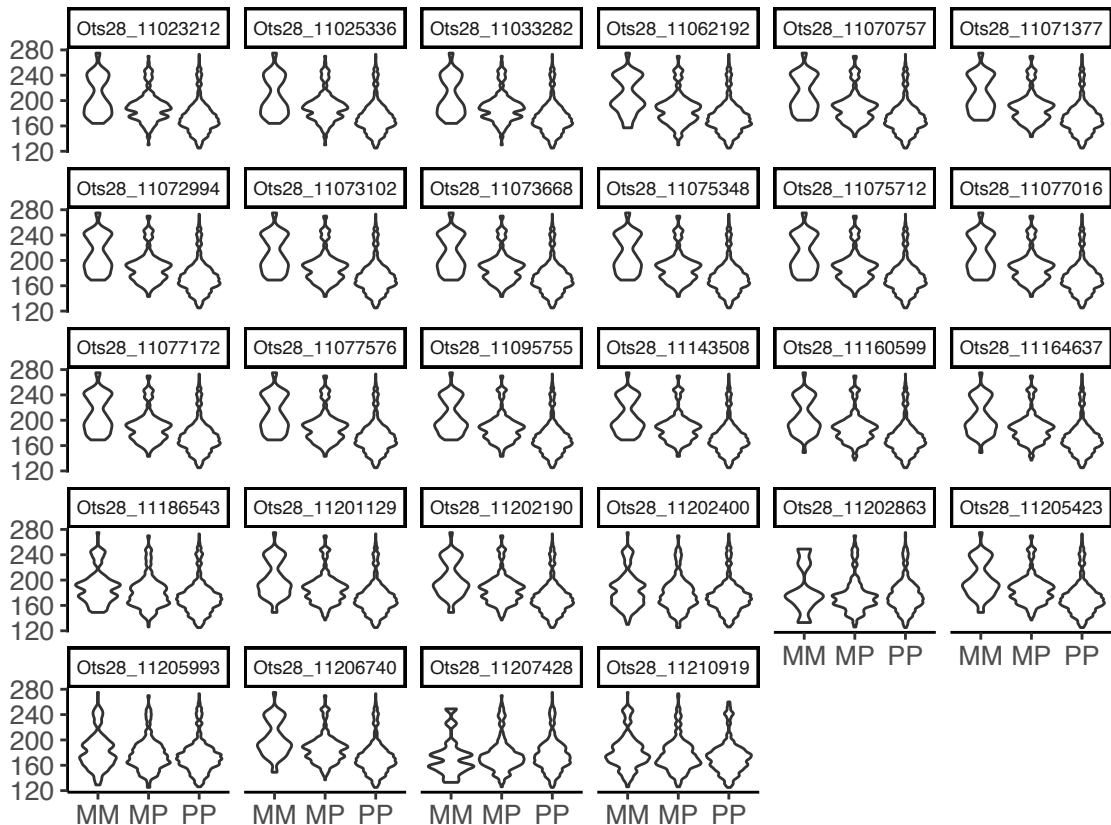

Genotype

Supplement: Supplementary file 7 — Fig S7 [file EVA-14-2273-s005.pdf]

Passage Day

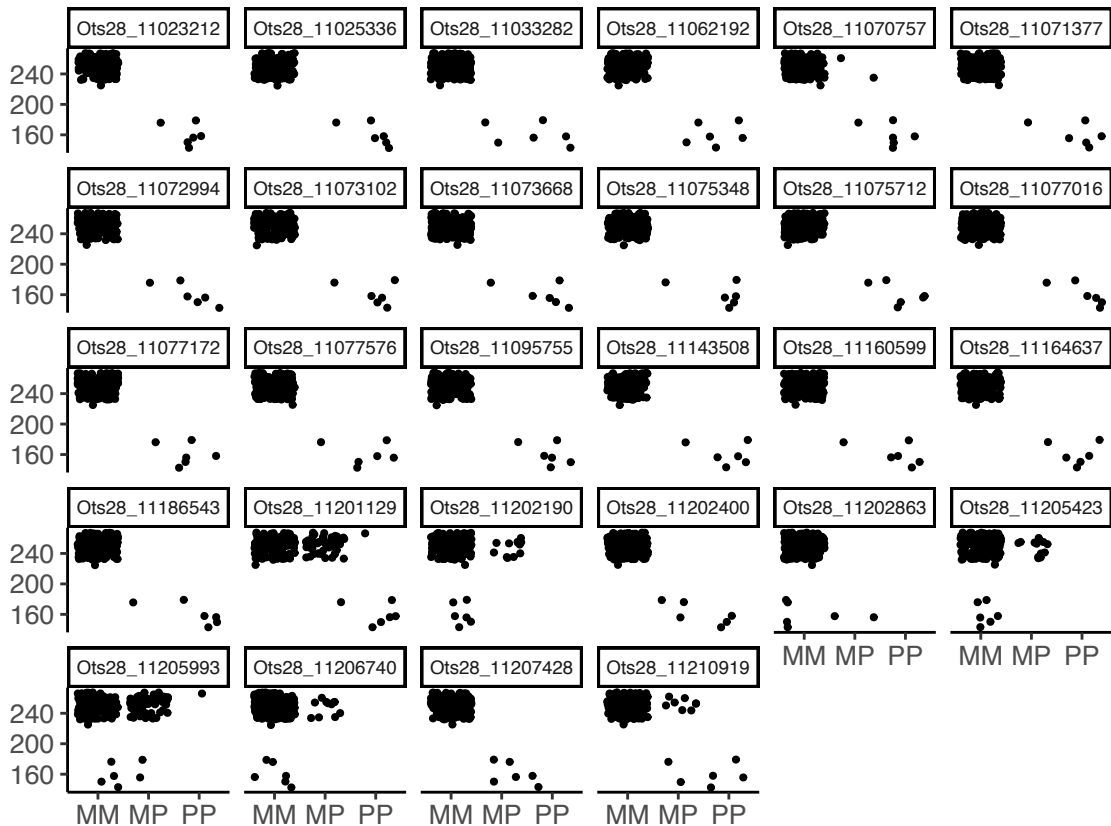

Genotype

Supplement: Supplementary file 8 — Fig S8 [file EVA-14-2273-s003.pdf]

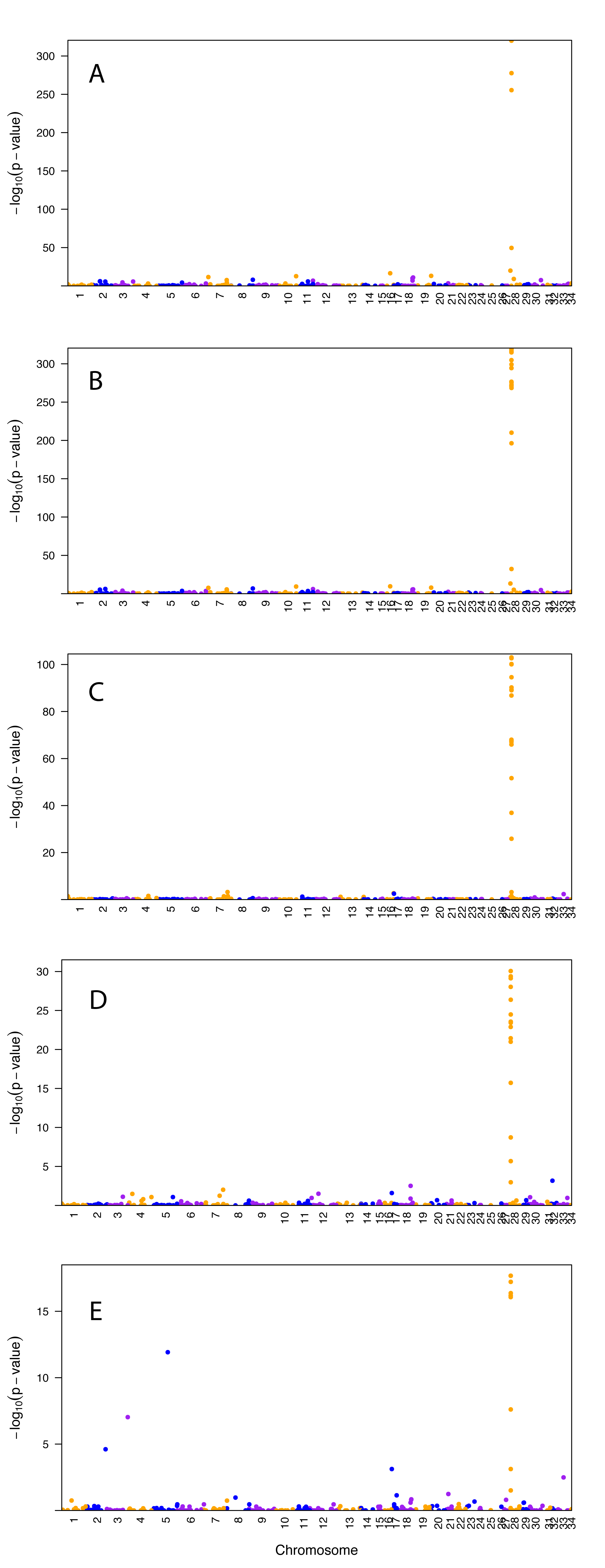

Supplement: Supplementary file 9 — Fig S9 [file EVA-14-2273-s008.tif]

Variance Explained

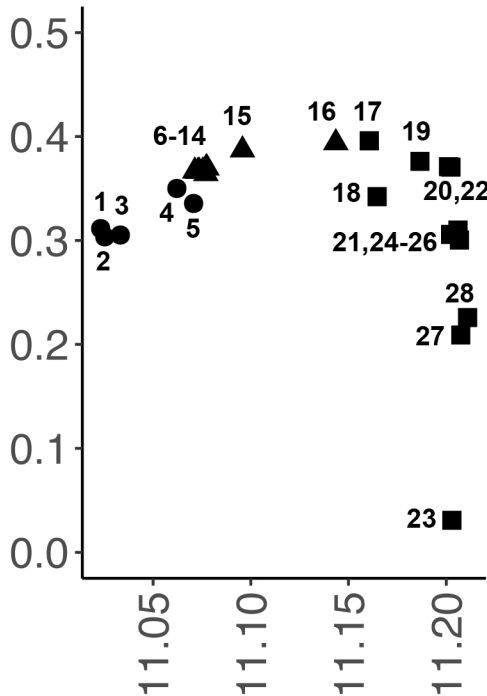

Position (Mbp)

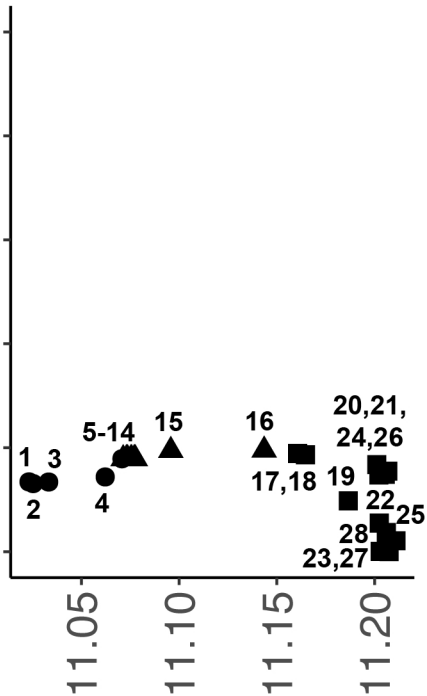

Supplement: Supplementary file 10 — Fig S10 [file EVA-14-2273-s012.pdf]

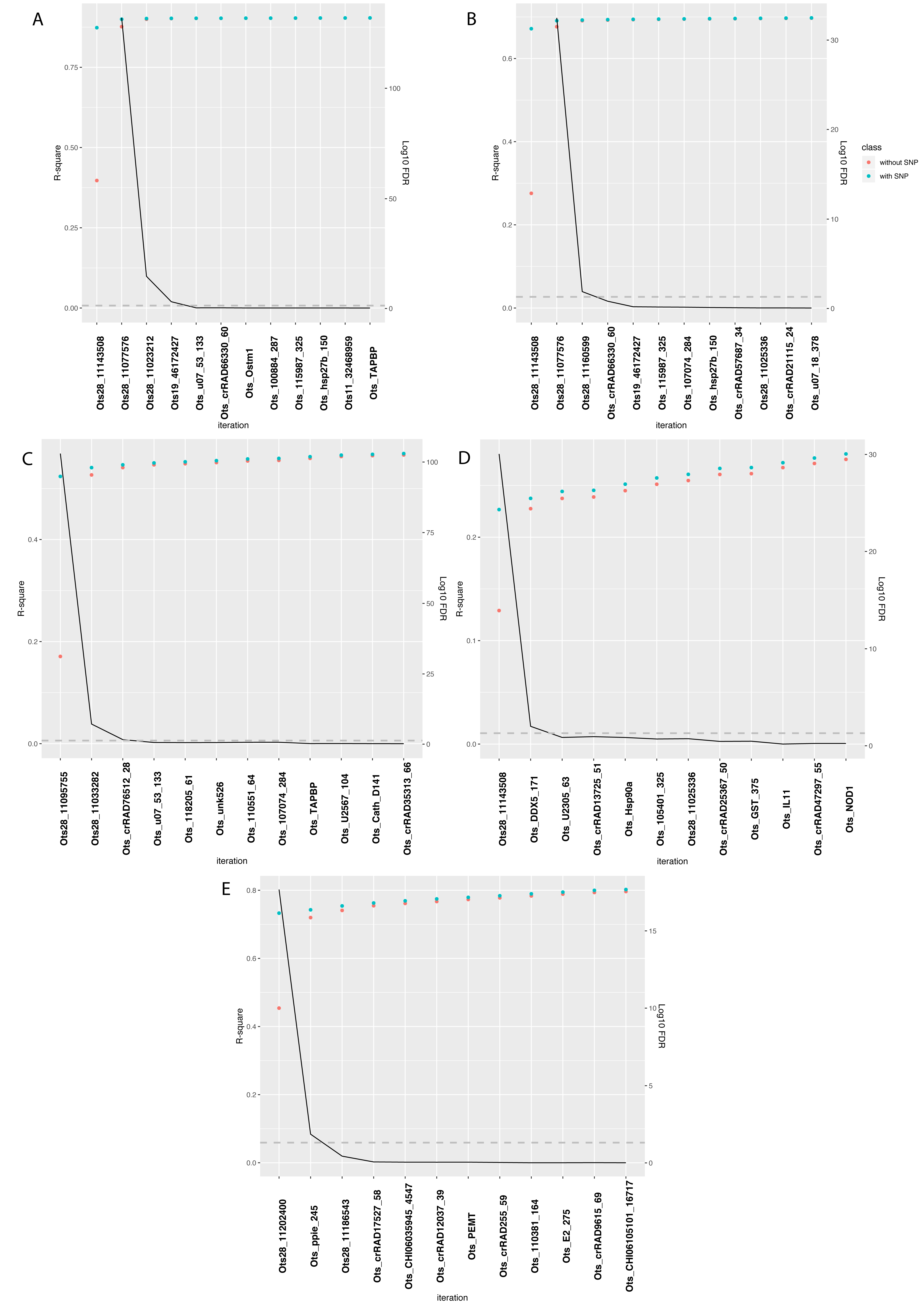

Supplement: Supplementary file 11 — Fig S11 [file EVA-14-2273-s009.tif]

Proportion

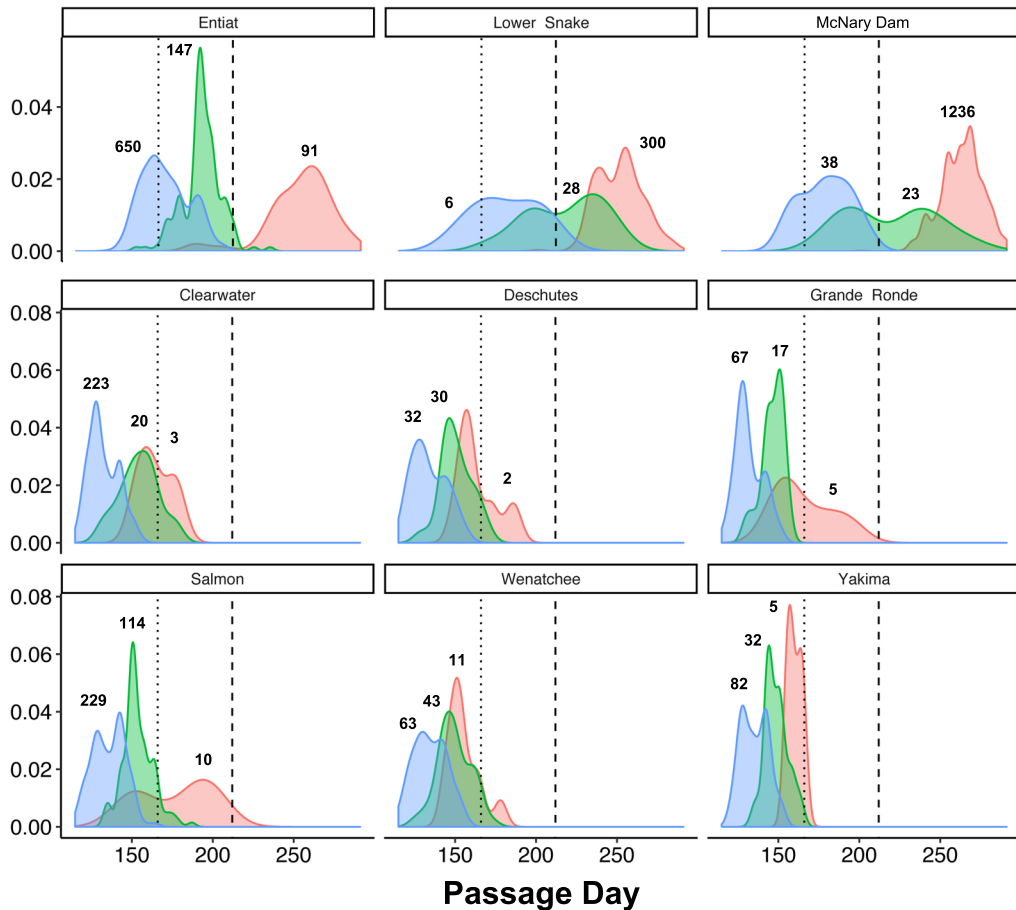

Supplement: Supplementary file 12 — Fig S12 [file EVA-14-2273-s004.pdf]
