# Supplementary material for: Genomic region associated with run timing has similar haplotypes and phenotypic effects across three lineages of Chinook salmon
Source: Evol Appl. 2021 Sep 1;14(9):2273–85. doi: 10.1111/eva.13290 (PMC8477596; doi:10.1111/eva.13290)
Supplement: Supplementary file 3 — Fig S3 [file EVA-14-2273-s016.pdf]

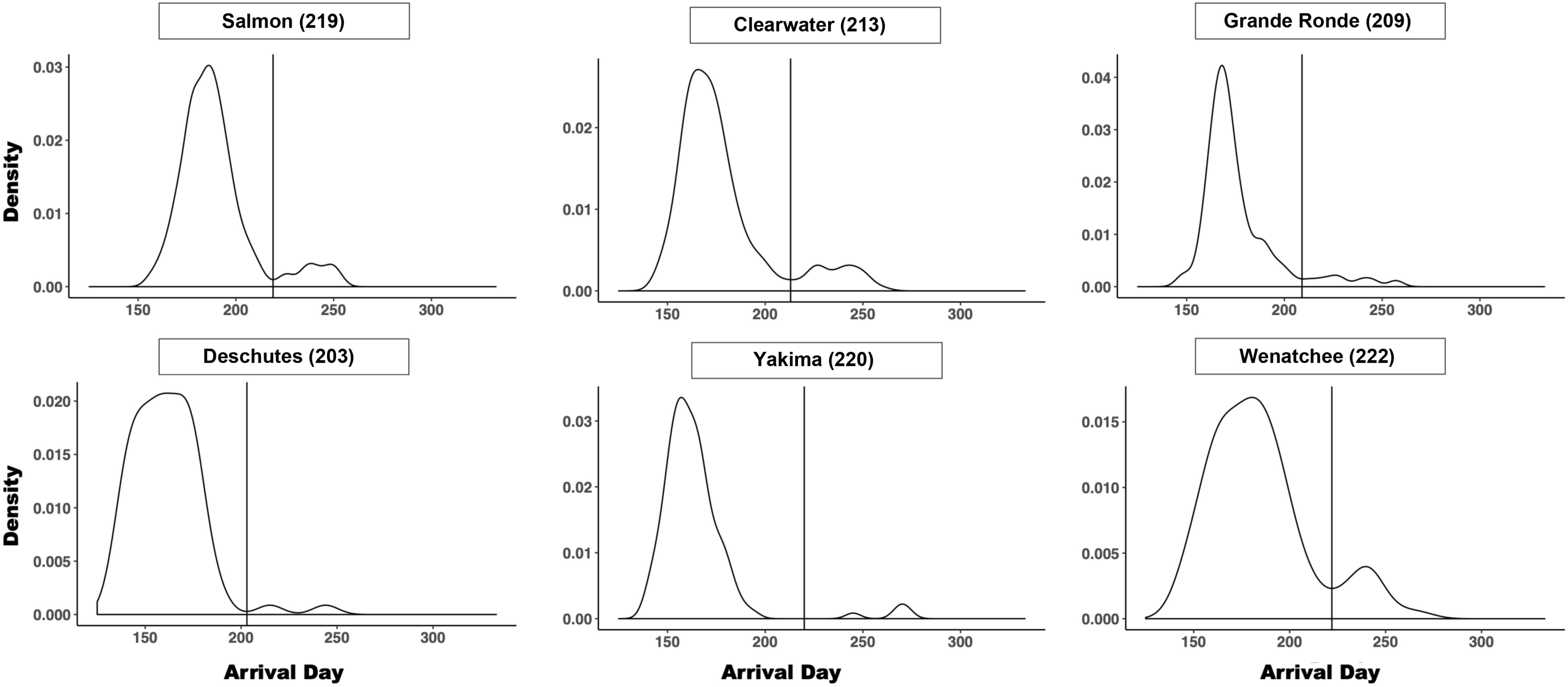

Salmon (219)
Clearwater (213)
Grande Ronde (209)
Deschutes (203)
Yakima (220)
Wenatchee (222)
Density
Arrival Day
0.00
0.01
0.02
0.03
0.04
0.000
0.005
0.010
0.015
0.020
150
200
250
300
