# Supplementary material for: Genomic region associated with run timing has similar haplotypes and phenotypic effects across three lineages of Chinook salmon
Source: Evol Appl. 2021 Sep 1;14(9):2273–85. doi: 10.1111/eva.13290 (PMC8477596; doi:10.1111/eva.13290)
Supplement: Supplementary file 6 — Fig S6 [file EVA-14-2273-s002.pdf]

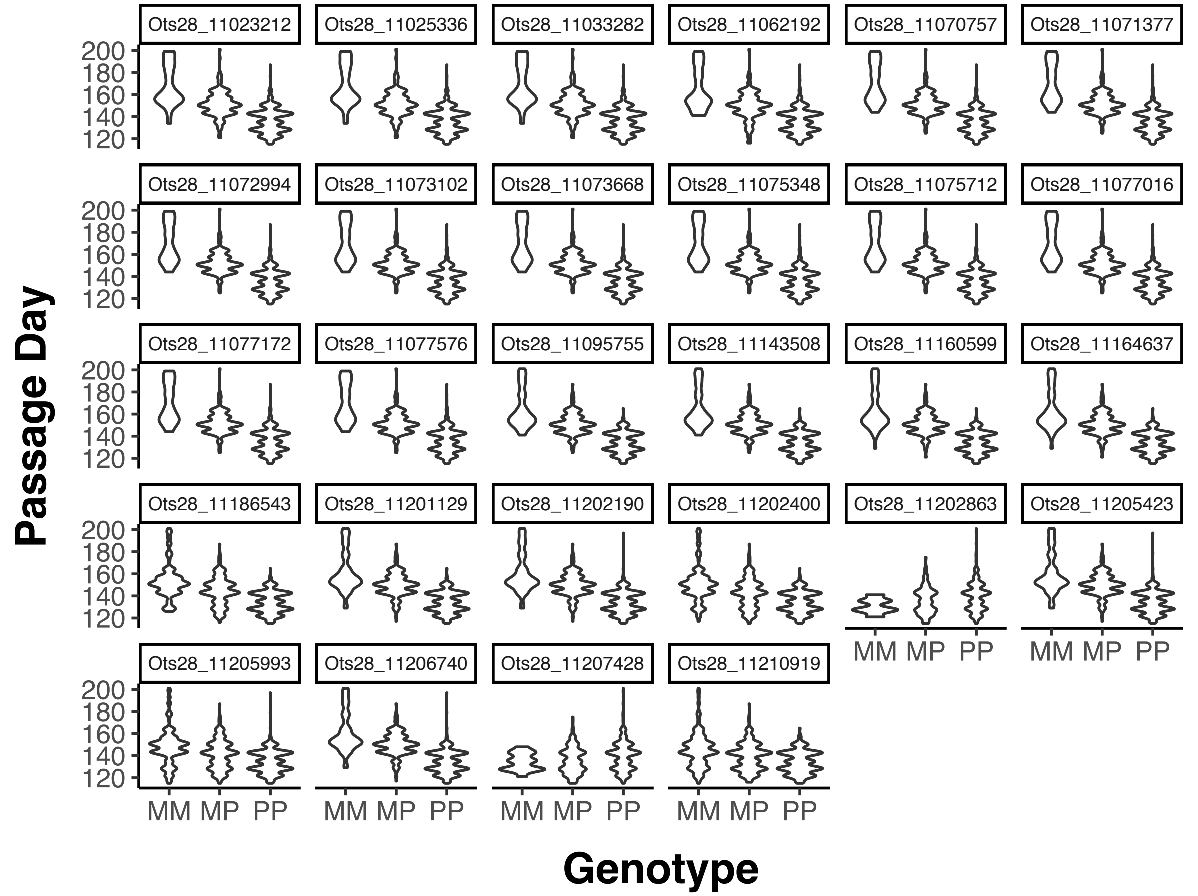

Ots28_11023212
Ots28_11025336
Ots28_11033282
Ots28_11062192
Ots28_11070757
Ots28_11071377
Ots28_11072994
Ots28_11073102
Ots28_11073668
Ots28_11075348
Ots28_11075712
Ots28_11077016
Ots28_11077172
Ots28_11077576
Ots28_11095755
Ots28_11143508
Ots28_11160599
Ots28_11164637
Ots28_11186543
Ots28_11201129
Ots28_11202190
Ots28_11202400
Ots28_11202863
Ots28_11205423
Ots28_11205993
Ots28_11206740
Ots28_11207428
Ots28_11210919
200
180
160
140
120
MM
MP
PP
Passage Day
Genotype
